# Centralized Energy Harvesting-Based TDMA Protocol for Terahertz NanoSensor Networks

**DOI:** 10.3390/s19204508

**Published:** 2019-10-17

**Authors:** Juan Xu, Jiali Kan, Yan Zhang

**Affiliations:** School of Electronics and Information Engineering, Tongji University Jiading Campus, Shanghai 201804, China; 15656963590@163.com (J.K.); zagyn_lss@163.com (Y.Z.)

**Keywords:** WNSNs, Terahertz, TDMA, energy harvesting, MDP

## Abstract

Terahertz wireless nano-sensor networks (WNSNs) are novel networks interconnecting multiple nano-devices by means of wireless communication. In this paper, a centralized energy harvesting-based time division multiple access (TDMA) protocol, called CEH-TDMA is proposed. This protocol examines the data transmission process from a global perspective, where the nano-controller regulates the channel access and allocates time slots for all nano-nodes. First, each nano-node sends the remaining energy and the number of packets in its data buffer to the nano-controller, and then, the nano-controller constructs a Markov decision process (MDP) model according to the state information of all nano-nodes, where the energy consumption and the number of transmitted packets in the entire network are considered as impact factors in designing the award function in the MDP model. Finally, a globally optimal slot allocation strategy is obtained, which maximizes the amount of packet transmission in the perpetual WNSNs.

## 1. Introduction

Nanotechnology enables the design and manufacture of nano-sensors with sensing, data storing, computing and communication capabilities [1]. These nano-sensors are able to sense events at the nano-scale, which is different from those in classical wireless sensor networks (WSNs). Wireless NanoSensor Networks (WNSNs) are new types of networks combing nanotechnology and sensor networks, which have broad application prospects in health monitoring, damage detection, biomedicine and military defense [2].

Recent simulation studies from [3] show that these nanosensors can communicate in Terahertz (0.1–10 THz) band using a new nano-material called graphene as parts of the transmission antenna. Terahertz band communication is considered to be the key technology to satisfy ultra-high-speed wireless communication, because it can provide very large transmission rate, up to Gb/s or even higher. Furthermore, nano-devices take advantage of the peculiarities of terahertz wave, such as the narrow beam and good directivity which can be used to detect and precisely position smaller targets [4]. On this basis, ref. [5] proposed a simple and effective modulation scheme of nano-devices called TS-OOK (Time Spread On-Off Keying), which is based on the exchange of femtosecond-long pulses spread in time. But in a TS-OOK communication system, the probability of collision increases with the increase of the number of transmitters [6]. Due to the high density of nodes in WNSNs, when multiple nano-devices send messages to the same target nano-node at the same time, if one symbol conflicts, it will cause conflicts in each symbol until the end of the first packet. There will be catastrophic collisions [7] that are unacceptable in many applications.

Considering the Terahertz band and characteristics of nano-devices in WNSNs, such as high propagation losses and very large distance-dependent bandwidth [7,8], new Media Access Control (MAC) protocols are required to regulate the channel access and transmission sequence between nodes. Recently, several MAC protocols have been proposed for WNSNs. Jornet et al. proposed and analysed a Physical Layer Aware MAC Protocol for Terahertz Electromagnetic Nanonetworks (PHLAME) [8]. To solve the energy consumption and reliability, PHLAME chooses the optimal value of code weight and repetition. A novel MAC protocol was proposed in [9] for cluster-based WNSNs, which is divided into two phases: the selection of a master node and the data transmission. An energy harvesting-aware and lightweight MAC protocol was proposed in [10], the protocol made the lifetime of WNSNs unlimited. Then, [11] introduced a Distributed Receiver-Initiated Harvesting-aware MAC for Nanonetworks (DRIH-MAC), which is designed to operate in a distributed network topology. But there are still hidden terminal problems in the distributed model. The Timing Channel for Nanonetworks (TCN) MAC protocol in [12] exploits the timing channel for transmissions. But the energy efficiency of TCN is not evaluated on simulation platforms. Rikhtegar et al. [13] proposed an energy efficient MAC protocol for Terahertz electromagnetic WNSNs (EEWNSN-MAC), which exploits the hierarchical structure and combines the contention free scheme, time division multiple access (TDMA) with a clustering algorithm for communication between nano-nodes. However, EEWNSN-MAC does not take the dynamic changes in transmitted packets of each nano-node and the energy harvesting system into consideration. Sami et al. [14] proposed a cooperative MAC protocol called cooperative cognitive TDMA (CC-TDMA) for cognitive networks, which guarantees the quality of service (QoS) required by the primary network. The unlicensed users obtain greater opportunity for data transmission, thus increasing their performance. However, the performance of the protocol in the terahertz band cannot be predicted. A load-aware dynamic TDMA protocol (LA-TDMA) [15], which realized the dynamic allocation of data transmission time slots on the basis of TDMA according to terahertz channel characteristics, number of nano-nodes and respective traffic volume of nodes. LA-TDMA ensures that data is transmitted without conflict, but it does not consider the priority difference of data. Cao et al. proposed the MAC protocol of individual domain network with high throughput and low delay [16]. Based on IEEE 802.15.3c [17], the high-throughput low-delay MAC (HTLD-MAC) protocol adopts the slot reservation mechanism based on channel quality and the adaptive confirmation mechanism. Nodes choose the block confirmation mechanism or immediate confirmation mechanism according to the channel quality, which can reasonably allocate slot resources, reduce data delay and improve network throughput. Mohrehkesh et al. proposed a MAC protocol based on receiving node initiation and energy acquisition, called RIH-MAC [18]. The main goal of this protocol is to reduce energy consumption. In addition, this protocol adopts the handshake process initiated by receiving node rather than the handshake process initiated by sending node. However, the new problem is that the protocol cannot solve the problem of hidden terminals in ad-hoc networks, and the energy collection process takes a long time.

Since a very limited amount of electricity can be stored in nano-batteries and it’s difficult to replace or charge the nano-batteries in most application scenarios [19], the energy limitation of nano-devices becomes one of the main challenges in the design of MAC protocols. Energy harvesting technology can continuously replenish energy for batteries, which makes sense in ensuring the sustainability of the system. A nano-scale energy harvesting system is proposed in [20], which converts the harvested vibration energy, sound energy and electromagnetic energy into electric energy. The distributed energy harvesting TDMA (DEH-TDMA) protocol in [21] introduces a piezoelectric nano-energy harvesting system due to the limited energy of nano-nodes. However, this protocol only makes decisions from the perspective of the nano-node itself and does not comprehensively consider the state information of other nodes in the network. Therefore, the decision results obtained by solving the MDP model are only the local optimal strategy.

In this paper, a centralized energy harvesting-based TDMA protocol, called CEH-TDMA is proposed. This protocol examines the data transmission process from a global perspective, where the nano-controller regulates the channel access and allocates time slots for all nano-nodes. The rest of this paper is organized as follows. Section 2 introduced the symbol model. Next, a Markov decision process (MDP) model for the proposed CEH-TDMA is built and an optimal slot strategy is solved in Section 3, and the establishment steps of CEH-TDMA is described in Section 4. In Section 5, we present simulation results for the performance of TDMA, LA-TDMA and DEH-TDMA based on average end-to-end delay, average remaining energy and number of transmitted packets. Conclusions are made in Section 6.

## 2. System Model

### 2.1. Network Model

CEH-TDMA adopts a star topology based on single-hop communication, which consists of a nano-controller with strong computational and processing capabilities and a number of common nano-nodes. The network topology of WNSNs can be represented by G=(V,E), where $V=\left\{v_{0},v_{1},\ldots ,v_{n}\right\}$ is the set of the nano-controller and all nano-nodes, $v_{0}$ is the nano-controller and E is the set of all single-hop links between nano-nodes.

The network model has the following assumptions:

- Self-powered nano-nodes are randomly distributed in the monitoring area, and the location of the nano-controller and nano-nodes are fixed.
- Nano-nodes can sense the remaining energy of their nano-batteries. All nano-nodes can harvest energy from the environment through the piezoelectric energy harvesting system. The energy of the nano-controller is not limited, and the data sensed by nano-nodes will be stored in the buffer queue.
- The energy consumed in data transmission is the main energy consumption during sensing, calculating and transmitting data, while the energy consumed in sensing and calculating data will be neglected.

### 2.2. Markov Decision Process

MDP is a decision-making model that can be widely used to describe the interaction between decision makers and the environment. The future state of the Markov process is only related to the current state and is independent of the historical state. Hence, the state transition probability and reward in the MDP model only depend on the current state and the action chosen by the decision maker. Similarly, in energy harvesting-based WNSNs, the current energy and packet state of nano-nodes are only related to the state of the last moment. Therefore, MDP can be applied to the design of MAC protocols which adopts the energy harvesting system, and the nano-controller serves as a decision maker in the MDP model.

### 2.3. Energy Harvesting Model

The main principle of energy harvesting is to convert some external energy sources such as vibrations in the surrounding environment or artificially generated ultrasonic waves into electrical energy. The maximum energy stored in the ultra-nano-capacitor $E_{max}=C_{cap}U_{g}{}^{2}/2$, where $C_{cap}$ is the total capacitance of the ultra-nano-capacitor and $U_{g}$ is the generator voltage. 

And the energy harvesting rate $\lambda_{e}$ [22] in Joule/second at which the ultra-nano-capacitor is charged can be written as:

(1) $$\lambda_{e}=\;f_{v}U_{g}\Delta Q(e^{-\frac{\Delta Q}{U_{g}C_{cap}}n_{cr}}-e^{-\frac{2\Delta Q}{U_{g}C_{cap}}n_{cr}}),$$

where $f_{v}$ is the vibration frequency of the external energy source, $n_{cr}$ is the number of cycles and ΔQ is the harvested charge per cycle. For a common vibration source, the energy harvesting process follows a Poisson distribution [22]. Therefore, the energy arrival within one frame of duration $t_{f}$ can be described as (2).

(2) $$\begin{array}{ll} f_{E}(x) & =\mathrm{Poisson}(harvested\;energy=x)\\ & =\left\{ \begin{array}{l} e^{-\lambda_{e}\cdot t_{f}}\frac{{\left(\lambda_{e}\cdot t_{f}\right)}^{x}}{x!},\;\mathrm{if}\;\;x\geq 0\\ 0,\;otherwise \end{array}\right. \end{array},$$

### 2.4. Packet Arrival Model

Poisson Process Model in [23] is used to describe the packet arrival in the buffer of nano-nodes, and the packet arrival process follows the principle of FIFO. Assuming that the average packet arrival rate is $\lambda_{p}$, the packet arrival within one frame of duration $t_{f}$ can be written as:

(3) $$\begin{array}{ll} f_{D}(y) & =\mathrm{Poisson}(packets\;arrival=y)\\ & =\left\{ \begin{array}{l} e^{-\lambda_{p}\cdot t_{f}}\frac{{\left(\lambda_{p}\cdot t_{f}\right)}^{y}}{y!},\;\mathrm{if}\;\;y\geq 0\\ 0,\;otherwise \end{array}\right. \end{array},$$

### 2.5. Energy Consumption Model

The main energy consumption is the energy consumed in receiving and transmitting data of nano-nodes. The energy consumption of receiving and transmitting per bit data in a THz communications system based on TS-OOK can be expressed as:

(4) $$E_{c}=\omega E_{ptx}+E_{prx},$$

where $E_{c}$ is the total energy expenditure of transmission and reception per bit data, $E_{ptx}$ and $E_{prx}$ respectively represent the energy expenditure of transmission and reception a single pulse. ω is related to the coding weight and usually set to 0.5 [6].

In order to reduce the algorithm complexity of introducing MDP model in MAC protocols, $E_{ptx}$ and $E_{prx}$ in (4) is respectively set to 1 pJ and 0.1 pJ when the communication range of nano-nodes is 0.01 m [6].

## 3. MDP Model for CEH-TDMA

**Figure 1:**
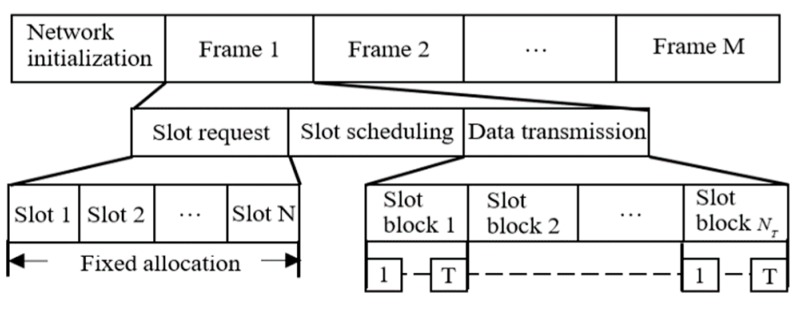
Frame Structure of CEH-TDMA.

The CEH-TDMA protocol adopts a frame structure shown in Figure 1. The nano-node informs the nano-controller of the status information including the number of data packets in the buffer area and the remaining energy quantity, and the nano-controller constructs a specific MDP model according to the information and solves the global optimal strategy, and then the result is broadcast to the nano-node. At this time, the nano-node obtains the decision result of the nano-controller from the time slot scheduling packet, and then occupies the corresponding time slot to transmit data. We will establish the system state, action space, state transition probability matrix and award function of the MDP model for the CEH-TDMA protocol respectively, considering the calculation complexity of the MDP model and in order to maximize the data transmission volume of the entire network, an approximate time slot allocation strategy is obtained by approximate solution.

### 3.1. System State Space

Since the nano-controller examines the data transmission process of WNSNs from a global perspective, the system state is the joint state of all nano-nodes. Then in frame f, the system state space is $S_{f}=\left(S_{f,1},\ldots ,S_{f,ni},\ldots ,S_{f,N}\right)$, where $S_{f,n_{i}}$ represents the state of the nano-node $n_{i}$, where N is the total number of nano-nodes in WNSNs.

In the $f_{th}$ frame, the state $S_{f}$ of the nano-node $n_{i}$ is the joint state consisting of the number of packets in the buffer $D_{f}$ and the remaining energy $E_{f}$, which can be expressed as:

(5) $$\begin{array}{l} S_{f,n_{i}}=\left(D_{f,n_{i}},E_{f,n_{i}}\right)\\ D_{f}\in \left\{0,\cdot \cdot \cdot ,D_{max}\right\},\;\;E_{f}\in \left\{E_{min},\cdot \cdot \cdot ,E_{max}\right\} \end{array},$$

where $D_{max}$ is the maximum number of packets in the buffer, $E_{\min}$ is the minimum energy required for normal operation of nano-nodes, which is set to the energy consumed in sending and receiving a packet, $E_{max}$ is the maximum energy stored in the nano-battery.

In order to facilitate the calculation, normalized energy is adopted, where the remaining energy of the nano-node is mapped to the number of packets that can be sent under the current energy constraint:

(6) $$NE_{f}=\left\lfloor (E_{f}-E_{min})/E_{tx}\right\rfloor ,\;\;\;\;\;\;NE_{f}\in \{0,\cdot \cdot \cdot ,N_{T}\},$$

(7) $$N_{T}=\left\lfloor (E_{max}-E_{min})/E_{tx}\right\rfloor ,$$

where $NE_{f}$, $N_{T}$ respectively represents the number of packets that the nano-node can send when the remaining energy reaches $E_{f}$ and the maximum $E_{max}$, $E_{tx}$ is the energy consumption of sending $N_{bit}$ packets. ⌊⋅⌋ is a rounding operation.

For self-powered nano-node $n_{i}$, the number of packets in the buffer and the remaining energy can be expressed as:

(8) $$D_{f+1}=\min(\max(D_{f}+S(\Delta t)-\alpha_{f}P_{f},0),D_{max}),$$

(9) $$E_{f+1}=\min(\max(E_{f}+H(\Delta t)-\beta_{f}P_{f}E_{tx},E_{min}),E_{max}),$$

where $D_{f+1}$ and $D_{f}$ respectively denotes the number of packets in the buffer at the beginning of the $f_{th}+1$, $f_{th}$ frame, $E_{f+1}$ and $E_{f}$ respectively denotes the remaining energy at the beginning of the $f_{th}+1$, $f_{th}$ frame. Δt is the time length of the $f_{th}$ frame, S(Δt) and H(Δt) is the number of packets sensed from the external environment and the energy acquired from the energy harvesting system during Δt, respectively. $\alpha_{f}$ and $\beta_{f}$ are both binary parameters, where $\alpha_{f}=\beta_{f}=1$ means the nano-node ni is active and participates in data transmission in the $f_{th}$ frame; otherwise $\alpha_{f}=\beta_{f}=0$. The number of transmitted packets is denoted by $P_{f}$, $P_{f}\cdot E_{tx}$ is the energy consumption of sending $P_{f}$ packets in the $f_{th}$ frame. The number of sensed packets $S(\Delta t)=\left\lfloor \lambda_{p}\cdot \Delta t\right\rfloor$, and the harvested energy $H(\Delta t)=\left\lfloor \lambda_{e}\cdot \Delta t\right\rfloor$.

### 3.2. Action Space

In the CEH-TDMA protocol, the nano-controller determines the channel access of each nano-node according to the state space established in the previous section. Therefore, the action space is a joint space including the action of each nano-node, denoted by $A_{f}=\left\{A_{f,1},\ldots ,A_{f,n_{i}},\ldots ,A_{f,N}\right\}$, where $A_{f,n_{i}}$ is the action space of one nano-node in the WNSNs. According to the above analysis of $A_{f,n_{i}}\in \left\{a_{0},a_{1}\right\}$ where $a_{0}=1$ indicates that the node in the current frame enters the dormant state due to lack of available energy or no data arrival in the buffer, $a_{1}=1$ indicates that the nano-battery has sufficient energy to transmit data.

### 3.3. State Transition Probability

In general, the data arrival and energy harvesting process of nano-nodes are independent in energy harvesting-based WNSNs. Therefore, it can be assumed that the state transition probability of packet and energy are also independent, so that the system state transition probability of nano-nodes can be obtained by solving the energy state transition probability and the packet state transition probability, respectively.

Firstly, the amount of data transmitted and energy consumption by nano-node $n_{i}$ when taking the appropriate action $A_{f}$ under its current state $S_{f}=\left(D_{f},E_{f}\right)$ can be written as:

(10) $$P(S_{f},A_{f})=\min(T,\min(D_{f},NE_{f})),$$

(11) $$E(S_{f},A_{f})=P(S_{f},A_{f})\cdot E_{tx},$$

(12) $$T=\min(D_{max},N_{T}),$$

where $P(S_{f},A_{f})$ and $E(S_{f},A_{f})$ respectively denotes the number of transmitted packets and energy consumption in the $f_{th}$ frame, $D_{f}$ and $NE_{f}$ respectively denotes the number of packets in the buffer and the number of packets that can be transmitted with the remaining energy $E_{f}$ in the current frame, T is the number of packets that can be transmitted in the slot block shown in Figure 1. In order that nano-nodes can send packets to the nano-controller as many as possible, the value of T is set to the maximum amount of data transmission in the current frame, which is jointly determined by $D_{max}$ and $N_{T}$, that is, the maximum number of packets in the buffer and the number of packets that can be sent when the remaining energy reaches the maximum.

The number of packets arriving in the buffer and the energy arriving in the nano-battery during state transition can be expressed as:

(13) $$d=\left\lceil D_{f+1}-D_{f}+P(S_{f},A_{f})\right\rceil ,$$

(14) $$e=\left\lceil (E_{f+1}-E_{f}+E(S_{f},A_{f}))/E_{tx}\right\rceil ,$$

where $P(S_{f},A_{f})$ and $E(S_{f},A_{f})$ respectively denotes the number of transmitted packets in (10) and the energy consumption in (11), the round-up operator ⌈⋅⌉ is adopted in the above two formulas since the number of packets and energy are all integers and nano-nodes are expected to transmit data as much as possible with sufficient energy in CEH-TDMA.

Substituting (13) into the packet arrival model described by (3), we can get the packet state transition probability $P_{e}(D_{f+1}|D_{f},A_{f_{i}})$.

Similarly, substituting (14) into the energy harvesting model described by (2), we can easily get the energy state transition probability $P_{e}(D_{f+1}|D_{f},A_{f_{i}})$.

So, the system state transition probability of the node can be expressed as the product of the packet state transition probability and the energy state transition probability [23]:

(15) $$\begin{array}{ll} P_{n_{i}}(S_{f+1,n_{i}}|S_{f,n_{i}},A_{f,n_{i}})= & P_{d,n_{i}}(D_{f+1,n_{i}}|D_{f,n_{i}},A_{f,n_{i}})\cdot \\ & P_{e,n_{i}}(E_{f+1,n_{i}}|E_{f,n_{i}},A_{f,n_{i}}) \end{array},$$

where $P_{d,n_{i}}(D_{f+1,n_{i}}|D_{f,n_{i}},A_{f,n_{i}})$ indicates the state transition probability of the packet of node $n_{i}$, and $P_{e,n_{i}}(E_{f+1,n_{i}}|E_{f,n_{i}},A_{f,n_{i}})$ represents the energy state transition probability of node $n_{i}$.

Since the nano-node needs to consume a certain amount of energy to transmit the state information and receive a slot scheduling packet broadcast by the nano-controller, and the calculation of the energy state transition probability involves the data transmission amount and corresponding energy consumption of the node in the frame, which can be defined as:

(16) $$E_{n_{i}}(S_{f},A_{f})=(N_{con}+P(S_{f},A_{f}))\cdot E_{tx}+N_{sch}\cdot E_{rx},$$

where $E_{n_{i}}(S_{f},A_{f})$ represents the energy consumption of node $n_{i}$ in frame f, $N_{con}$ and $N_{sch}$ represent the state information and the number of bits of the slot scheduling packet, respectively, $P(S_{f},A_{f})$ represents the amount of transmitted data in the frame f, $E_{tx}$ and $E_{rx}$ respectively represent the transmission and reception energy consumption of per bit.

It is assumed that each nano-node in WNSNs is individually aware of signals from the external environment, so the data arrival between nodes is independent of each other. Since the energy harvesting rate is strongly related to the vibration source and the environment, the energy harvesting process between nano-nodes has a certain correlation. However, CEH-TDMA aims to obtain a dynamic TDMA protocol based on the number of packets in the buffer and the remaining energy allocation slots, studying the correlation of energy harvesting rates requires a large amount of field test data, so the CEH-TDMA protocol assumes the process of collecting energy from the external environment by the nano-nodes is independent of each other, so that the energy state transitions between the nodes are also independent of each other. The system state is the joint state space composed of the states of all the nano-nodes, so that the system state transition probability is:

(17) $$P(S_{f+1}|S_{f},A_{f})=\overset{N}{\underset{n_{i}=1}{∐}}P_{n_{i}}(S_{f+1,n_{i}}|S_{f,n_{i}},A_{f,n_{i}}),$$

### 3.4. Award Function

In order to maximize the network throughput and reduce the energy consumption by solving the established MDP model, the award function of each nano-node can be written as:

(18) $$R(S_{f,n_{i}},A_{f,n_{i}})=\frac{P(S_{f,n_{i}},A_{f,n_{i}})}{\lambda_{p}\cdot t_{f}}-\frac{E(S_{f,n_{i}},A_{f,n_{i}})}{E_{c-max}},$$

where $R(S_{f,n_{i}},A_{f,n_{i}})$ is the award function obtained after nano-node $n_{i}$ takes action $A_{f,n_{i}}$ under stat $S_{f,n_{i}}$, $\lambda_{p}$ is the average packet arrival rate, $t_{f}$ is the length of a frame, $\lambda_{p}\cdot t_{f}$ is the average number of packets arriving within a frame, $P(S_{f,n_{i}},A_{f,n_{i}})$ and $E(S_{f,n_{i}},A_{f,n_{i}})$ are respectively the amount of transmitted data and energy consumption within the current frame and is calculated by (10) and (11), respectively, $E_{c-max}$ represents the maximum energy consumption for sending and receiving packets in the current frame. In addition, the normalized amount of transmitted data and energy consumption are adopted to analyze the award function.

The system award is the sum of the each nano-node’s award, specifically expressed as:

(19) $$R(S_{f},A_{f})=\sum_{n_{i}=1}^{N}R(S_{f,n_{i}},A_{f,n_{i}}),$$

where $R(S_{f,n_{i}},A_{f,n_{i}})$ represents the income function obtained by the behaviour of the nano-node $n_{i}$ taking the behaviour $A_{f,n_{i}}$ in the state $S_{f,n_{i}}$.

### 3.5. Approximate Solution of MDP Model

The time slot allocation strategy of the CEH-TDMA protocol is to establish and solve the MDP model according to the real-time state of each nano-node by the nano-controller, and finally determine the global optimal channel access mode. The nano-node only needs to know the decision result from the time-slot scheduling package broadcast by the nano-controller instead of participating in the decision-making process, which greatly reduces the calculation and memory pressure of the nano-node. However, the protocol needs to consider the state information of all nodes in the network, which increases the complexity of the MDP model built above. Hence we will propose an approximate solution algorithm to reduce the computational complexity, so that the CEH-TDMA protocol can be applied to a wide range of practical scenarios.

The nano-controller can count the number of source nodes $N_{T}$ in the current frame f under the condition of the known decision behavior space $A_{f}$, and set the number of time slot blocks in the data transmission phase to $N_{T}$. In the frame structure shown in Figure 1, in the data transmission phase, the number of time slot blocks varies with the number of source nano-nodes, and each time slot block contains T time slots and remains unchanged. Considering that in WNSNs based on energy harvesting, the amount of data transmitted by a nano-node in the current frame is limited by its residual energy, so a measure function is defined to determine time slot allocation during the data transfer phase.

(20) $$m_{n_{i}}=\min\left(D_{f,n_{i}},\left|E_{f,n_{i}}-E_{con}\right|/E_{tx}\right),$$

where $m_{n_{i}}$ represents the number of data packets that the nano-node $n_{i}$ wants to transmit in frame f, and is also the basis for the nano-controller to dynamically allocate time slots. $D_{f,n_{i}}$ and $E_{f,n_{i}}$ are the number of packets and residual energy sent by the nano-node to the nano-controller in the slot application phase, $E_{con}$ indicates the energy consumption of the nano-node transmitting status information and the receiving time slot scheduling packet, and $E_{tx}$ indicates the transmission energy consumption per unit bit.

Based on the time slot measurement function defined above and the greedy solution algorithm, we propose a method shown in Algorithm 1 for approximate solving the MDP model. First, the nano-controller can obtain their status information when they successfully receive the time slot request from the nano-node. Then, the time slot measure value of each node is calculated according to the formula (20), and the descending order is performed. Then, for a nano-node with a slot measurement value of 0, the nano-controller makes a decision that the time slot is not allocated, and the other nodes occupy *T* time slot blocks in descending order of the time slot measurement value, thereby ensuring a large amount of data and the node with higher remaining energy preferentially transmits data. Finally, the nano-controller broadcasts the decision behavior set and the slot allocation information to all the nano-nodes in the form of a time slot scheduling packet, and records the system state and behavior space at this time, so that the next time the same node state occurs, the nano-controller can directly obtain the corresponding decision result by searching, thereby eliminating the process of solving.


**Algorithm 1: Approximate solution for centralized MDP**
**input:** buffer and energy level of each nanonode $S_{f,ni}=\left(D_{f,ni},E_{f,ni}\right),n_{i}\in \left\{1,2,\cdot \cdot \cdot ,N\right\}$ **output:** a set of actions $A_{f}=\left\{A_{f,1},\ldots ,A_{f,ni},\ldots ,A_{f,N}\right\}$ compute slot measuring function $m=\left\{m_{1},m_{2},\ldots ,m_{N}\right\}$ according to Equation (20) sort m in descending order, enabling $m_{i}>m_{i+1},\forall i\in \left\{1,2,\cdot \cdot \cdot ,N\right\}$ **for each** element $m_{i}$ in m
**do**  **if**
$m_{i}>0$    **then**
$A_{f,ni}\leftarrow 1$  **else**
$A_{f,ni}\leftarrow 0$  **end if** **end for** **return**
$A_{f}=\left\{A_{f,1},\ldots ,A_{f,ni},\ldots ,A_{f,N}\right\}$


The above approximate solution algorithm needs to calculate the slot measure values of all nano-nodes and sort them in descending order, so the algorithm complexity can be expressed as O(NlogN+N). When N≥10, the algorithm complexity is O(NlogN), when 1<N<10, the algorithm complexity is O(N).

## 4. Establishment Steps of CEH-TDMA

**Figure 2:**
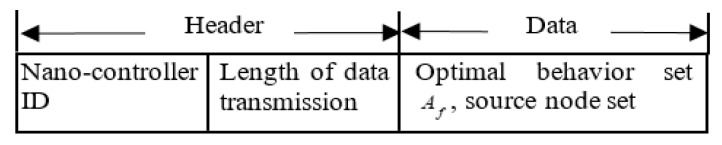
Time Slot Scheduling Packet Structure of CEH-TDMA.

CEH-TDMA includes four phases: network initialization, time slot application, time slot scheduling, and data transmission. The difference is that the nano-controller dynamically allocates time slots according to the joint state information composed of the number of data packets in the nano-node buffer area and the remaining energy. The specific establishment process is as follows:

1. The network initialization is mainly used to control clock synchronization and link establishment between the nano-controller and the nano-nodes. The nano-controller broadcasts the length of the single time slot and the total number of time slots in the slot application phase to the nano-nodes.
2. The nano-nodes occupy their respective time slots in the time slot application phase to send their real-time status information $S_{f,n_{i}}=\left(D_{f,n_{i}},E_{f,n_{i}}\right)$ to the nano-controller.
3. After receiving the state information of all nano-nodes, the nano-controller solves the behavior set corresponding to the node state $A_{f}=\left\{A_{f,1},\ldots ,A_{f,ni},\ldots ,A_{f,N}\right\}$ and the node ID set mapped to the $N_{T}$ time slot blocks in the data transmission phase based on the approximate solution algorithm described in Figure 1, and finally the decision result is reported to all nano-nodes in the form of a time slot scheduling packet. The slot scheduling packet structure is shown in Figure 2.
4. The nano-node gets the decision result of the nano-controller from the received time slot scheduling packet. If the behavior corresponded to the node $n_{i}$ is $A_{f,ni}=0$, then the node enters into the sleep state whose duration is tr, the length of the data transmission phase. If $A_{f,ni}=1$, the node accesses the channel according to the set of source node ID mapped to the slot block in the scheduling packet, and then transmits valid data.

## 5. Simulation Analysis

The simulation analysis measures the performance of the proposed protocol from three aspects: average end-to-end delay, average node residual energy and packet transmission amount. The packet transmission amount is defined as the total number of packets received by the nano-controller at the end of the simulation.

### 5.1. Parameter Settings

Set the simulation scene to a two-dimensional rectangle with 1 cm×1 cm in size, where the only one nano-controller located in the center of the region and 100 nano-nodes are independent and randomly distributed. The data transmission rate and communication range of each nano-node is set as 1 Mbps and 0.01 m, respectively. In addition, femtosecond-long pulse-based TS-OOK is adopted in the physical layer, the packet size and $D_{max}$ in (8) is respectively set as 128 bits and 5.

The piezoelectric energy harvesting system is adopted by all nano-nodes, where the voltage of the collection device $U_{g}$ is set to 0.42 V, the total capacity of the nano-capacitor $C_{cap}$ is set to 9 nF, hence the maximum energy of the nano-battery this time $E_{max}$ is 800 pJ. The amount of electricity collected per cycle ΔQ is set to 6 pC, the number of packets that can be sent when the stored energy reaches maximum $N_{T}$ from (7) is 11, the minimum energy $E_{\min}$ and the initial energy of each nano-node is set to 80 pJ and 800 pJ, respectively, the energy harvesting rate $\lambda_{e}$ in (2) is about 17 pJ/s when the vibration frequency of the external energy source $f_{v}$ is set to 50 Hz.

The state information sent by the nano-node is 8 bits, and the slot scheduling packet length is 200 bits. Therefore, the control energy consumption $E_{con}$ in (20) is set to 28 pJ. In the simulation, the fixed data packet generation interval is 10s, and study the changes in network performance with energy harvesting rate of the nano-node. The settings of the network parameters are summarized in Table 1.

### 5.2. Simulation Results

#### 5.2.1. Average End-to-end Delay

**Figure 3:**
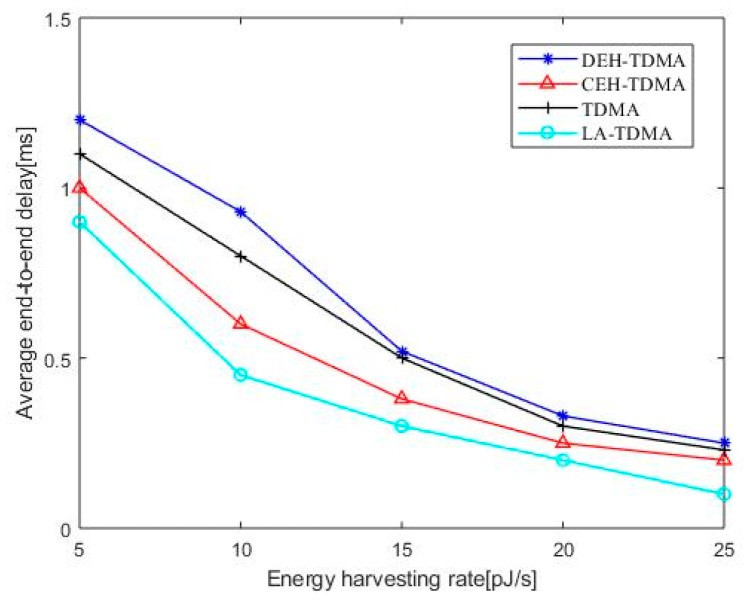
Average end-to-end delay as a function of energy harvesting rate.

Figure 3 is an end-to-end delay comparison of four MAC protocols. As can be seen from the figure, the end-to-end delay decreases as the nano-node energy harvesting rate increases. This is because the nano-battery is charged faster when the energy harvesting rate is increased, which greatly reduces the probability that the node cannot complete the data transmission due to lack of energy, thereby shortening the waiting time of the data packet in the buffer area, thereby reducing the average end-to-end delay and increasing the data transmission amount of the network.

As can be seen from the Figure 3, the end-to-end delay of CEH-TDMA is less than DEH-TDMA. Although the larger system state space causes the CEH-TDMA protocol to make channel access decisions longer than DEH-TDMA, CEH-TDMA determines the number of time slot blocks included in a frame based on the data and energy states of the nano-nodes and assigns time slots based on the priority of data transmission, so that nodes with larger data volume preferentially transmit data, such a dynamic time slot allocation method solves the problem of time slot wasting in DEH-TDMA, thereby reducing the average end to end delay. Since LA-TDMA adopts a dynamic slot allocation scheme where no time slots are allocated to idle nano-nodes while more time slots are provided for those data burst nano-nodes, LA-TDMA outperforms other protocols in average end-to-end delay.

#### 5.2.2. Average Remaining Energy of Nano-Nodes 

**Figure 4:**
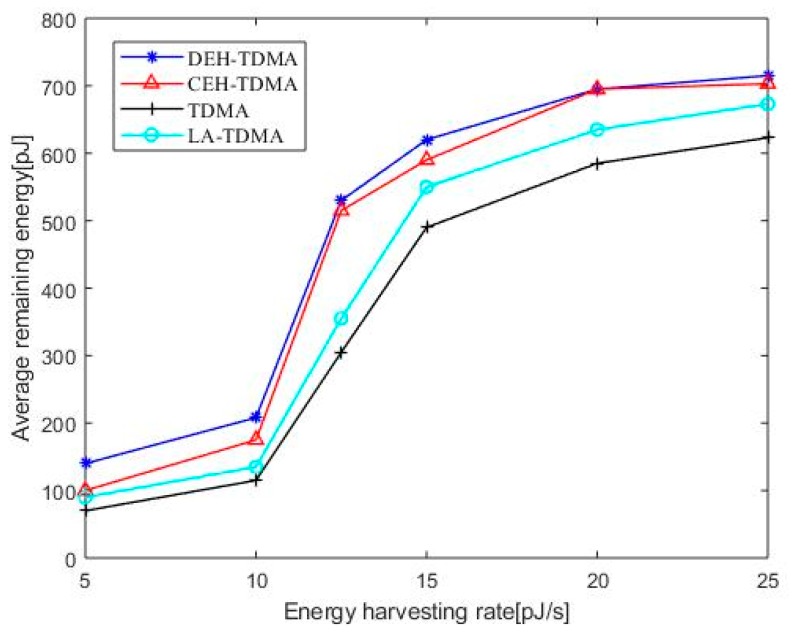
Average remaining energy as a function of energy harvesting rate.

Figure 4 shows the average residual energy comparison of four protocols. It can be seen that as the energy harvesting rate increases, the average residual energy of the nano-nodes in both protocols increases, because when the energy harvesting rate is low, the nano-nodes have less residual at the beginning of each frame. If the data is transmitted in this frame, and there is no supplemental energy available during this time, it will consume a large part of the energy; when the energy harvesting rate is gradually increased, the time for the nano-node to collect energy is greatly shortened, and the energy consumed by the data transmission can be compensated in time. 

Figure 4 shows that DEH-TDMA and CEH-TDMA outperform TDMA and LA-TDMA in terms of average remaining energy, DEH-TDMA and CEH-TDMA have little difference in the average residual energy of the nodes because the self-powered nano-nodes in the network are energy-compensated.

#### 5.2.3. Amount of Transmitted Packets 

**Figure 5:**
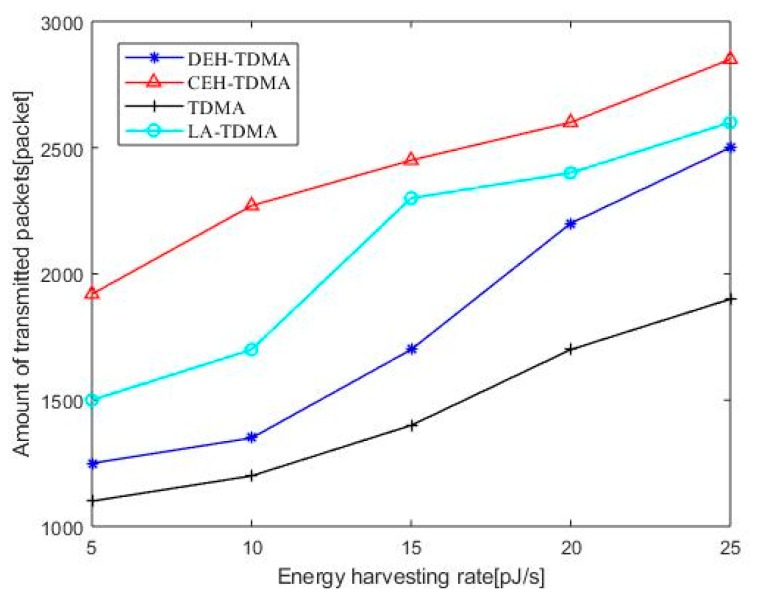
The number of packets transmitted as a function of energy harvesting rate.

Figure 5 shows the comparison of packet transmissions amount for four protocols. As can be seen that the packet transmission amount increases as the node energy harvesting rate increases, because the speed of energy harvesting can reduce the probability that the node cannot complete the data transmission due to lack of energy.

As can be seen from the Figure 5, CEH-TDMA is superior to LA-TDMA, DEH-TDMA and TDMA in packet transmission because CEH-TDMA protocol, the nano-controller arranges the nano-nodes with more buffered data packets and sufficient energy according to the sorted time slot measurement values to preferentially transmit data. This provides a buffer period for energy or data arrival for nano-nodes that are temporarily lacking energy or no packets in the buffer area, thereby increasing slot utilization and packet transmission amount throughout the network.

## 6. Conclusions

This paper examines the entire WNSNs from the perspective of the nano-controller, and proposes a centralized TDMA protocol based on energy harvesting, the proposed protocol determines the number of time slot blocks included in a frame based on the data and energy states of the nano-nodes and assigns time slots based on the priority of data transmission. The simulation results prove that the proposed CEH-TDMA has obvious advantages over the other three protocols in terms of number of transmitted packets. Moreover, its performance is almost as good as DEH-TDMA’s in terms of average remaining energy, and second only to LA-TDMA’s in terms of average end-to-end delay. The proposed protocol can maximize the amount of packet transmission in the perpetual WNSNs which can be used in in vivo WNSN to continuously monitor human health in real time. Therefore, our future work will focus on building human health monitor system by establishing in vivo nano-networks and designing suitable MAC protocols.

## Figures and Tables

**Table 1:** Parameter settings.

Simulation Parameters (unit)	Numerical Value
simulation scenario (cm^2^)	1 × 1
number of nano-nodes	100
number of nano-controller	1
physical layer pulse width (fs)	100
physical layer pulse interval (ps)	10
nano-node communication range (m)	0.01
data transmission rate (Mbps)	1
packet length (bit)	128
maximum packet	5
nano-node initial energy (pJ)	800
maximum energy	11
length of a single time slot (ms)	0.2
energy harvesting rate (pJ/s)	5~25
packet generation interval (s)	10
simulation time (s)	300
